# Supplementary material for: The impact of anthropometric characteristics on isometric trunk muscle endurance tests: A reliability and performance analysis
Source: PLoS One. 2025 Jun 3;20(6):e0324787. doi: 10.1371/journal.pone.0324787 (PMC12132975; doi:10.1371/journal.pone.0324787)
Supplement: Attachment — Submitted filename: Response to reviewers.docx [file pone.0324787.s003.docx]

The authors would like to thank the Reviewers and Editors for their advice and recommendations. We believe that the manuscript is stronger as a result of their comments.

The authors’ responses to each comment are bolded, and changes in the manuscript are presented in red.

**Reviewer #1: “Anthropometric characteristics impact the participant’s performance in popular isometric trunk muscle endurance tests”**

The Authors are commended for completing a considerable amount of high-quality work. I think the results of this investigation will contribute to advance knowledge on trunk testing. In this perspective, this work has potential for becoming a foundational piece for future translational studies using these tests in patient populations.

Some minor comments are presented below for the Editor’s and Authors’ consideration.

INTRO

L85-87 I suggest rephrasing a bit the following “questions such as: Which anthropometric variables have a greater impact on the performance of these tests? Could anthropometric differences between sexes affect these test scores? etc.”

**We have rephrasing the sentence… “including which anthropometric variables have a greater impact on test performance and the potential impact of sex-based differences in test scores”**

L97 The Authors may consider to:

1) State a specific hypothesis (and what they would anticipated as expected result from this investigation)

2) If and how more knowledge on this topic would benefit the scientific community and potential users of trunk control exercise testing

3) Who would care the most about new findings on this issue

**We have included this sentence at the end of the introduction “Based on previous studies (12–18), individuals´ anthropometric characteristics will have an impact on the isometric trunk muscle endurance test performance. These findings could improve the use and interpretation of these trunk muscle endurance field tests in clinical and sport settings”.**

METHODS

L100 and foll. The Authors may consider to better describe their cohort, e.g., how this sample was recruited, the level of physical activity, if they were from sampled from the general population or from university students’ populations, if there were athletes, etc. It is important to clarify the sample under study, also for replicability purpose.

**We have better describe our cohort including information about the type of population and physical activity performed “… with a regular practice of 2-5 times a week of 60-120 min per day of moderate physical activity for a total of 120-300 min per week voluntarily participated in the study. The participants were recruited from a university student´ population and were recreative athletes (i.e. soccer, gymnastics, basketball, running, etc).”**

L100 forty-five participants: was sampling stopped because you reached a predefined size of the cohort? Any a priori power analyses predefining an expected difference? Please, clarify

**Authors: We did not calculate a priori power analysis. Following, the reviewer´s suggestion we have included a post hoc power analysis.**

DISCUSSION

Overall, the discussion read well even though in some instances it includes results or data repetition which I would suggest deleting.

L343 “Between-sex analysis of the isometric trunk muscle endurance test scores”. I truly appreciated this paragraph, which discussed the findings in a physiologically driven manner. I wonder whether the previous sections would benefit from more physiological rather than biomechanic-only reasoning

**Authors: For the discussion of the data we have tried to use both results from previous studies and biomechanical principles and criteria. Whenever we have considered it appropriate, we have discussed the results in a more physiological manner. In response to your comment, in the section *Correlational analysis between the anthropometric measurements and the isometric trunk muscle endurance test scores*, we have included some sentences where we interpret some results based on the differences in muscle morphology and function:**

**“Moreover, anthropometric differences, such as a larger biileocrestal breadth, may change muscle fiber directions and traction angles in the trunk, hip and/or shoulder joints, impacting the function of the muscles that are activated to counteract gravitational torque during the performance of these holding tests. Further research is needed to understand the effect of participants’ anthropometric differences on muscle activation during these and other trunk muscle endurance tests, as well as the relationship of these changes in muscle function with test scores.”**

L377 The manuscript needs to be re-read carefully for some minor grammar errors and typos, such as “specially” at this line.

**Authors: The manuscript has been submitted to Proof-Reading-Service.com for editing and proofreading.**

Study limitations

I would suggest running a post hoc power analysis to see how much power you put together and if the sample is really limited as you acknowledged in the limitations.

**A post-hoc power analysis has been performed and included in manuscript.**

The software G*Power 3.1 (v3.1, University of Düsseldorf, Germany) was used to compute the pos hoc achieve power for the relationship between anthropometric variables and trunk endurance tests scores with the following parameters: males= -0.379 <r< 0.703; α= 0.05; total sample size= 27. Calculations suggested a statistical power of 1–β= 0.637 to 0.996; females= 0.493 <r< 0.747; α= 0.05; total sample size= 18. Calculations suggested a statistical power of 1–β= 0.698 to 0.958.

**The sample size is a limitation to elaborate normalized normative data that would allow to categorize the physical condition of the participants in a more realistic and adjusted way as we indicated in limitation section, but in general not for statistical analysis.**

Conclusions

I wonder whether this section could also include some elaboration on the potential clinical relevance of these tests and study findings for clinicians and their clients.

**Authors: We have included a practical application section which shows an example of two participants and the implications of our results for trainers and clinicians.**

Thank you for the opportunity to revise this fine paper.

**Reviewer #2:** The authors have submitted a manuscript in which they try to link anthropometric values with test performance of three different, but widely used trunk muscle endurance tests. Additionally the investigation also contains reliability data as one part of the investigation.
Major
Part one (reliability investigation)
For me it's somehow surprising that the anthropometric values at least partly were less reliable than the values for the tests. This does not speak for exact anthropometric measures, therefore questioning the whole investigation. Question: what anthropometric values were used for the correlation analyses?

**Authors: As we indicated in page 8; line 168: “Only those variables that obtained a moderate-to-excellent level of relative reliability (i.e., ICC > 0.60) were used to perform Pearson correlation analyses (r) between them”**

**We have revised our results and some reliability data of anthropometric variables were wrong. We agree with you that it can be somehow surprising that the anthropometric values at least partly were less reliable than the values for the tests and it could be also surprising for the readers. In addition, we appreciate your comment because it is really that it is not well explained in the manuscript or can be a bit confusing and therefore you and the readers could think that we do not have measured the anthropometric characteristics in an accurate way. But this is not true.**

**These are a brief definition for relative and absolute reliability. The relative reliability represents how well the rank order of participants in one trial is replicated in the second trial (normally evaluated with the ICC) while the absolute reliability is the degree to which repeated measurements vary for individuals (normally evaluated with the SEM).**

**If we analyze the reliability of the biacromial breadth in females we show that the SEM is 2.1% and ICC=0.73. This means that the error is only 0.75 cm for someone whose biacromial breadth is 37.5 cm (the females sample mean). If the sample is very homogeneous (as in our case), the ICC could be low because it is very difficult to replicate the same rank order between sessions, because the small SEM (0.75 cm) can do that participants change the rank order.**

**The logarithmic transformation of data was used in the reliability analysis to reduce the bias derived from the non-uniformity of the error and also in the correlation analysis** **to ensure the normality and homoscedasticity assumption.**

**The data of reliability of the table 1 and 2 refer to original data shown in the tables.**

What do the authors mean when using the term "relative reliability?

**- The relative reliability represents** **the** consistency of rank order of participants from test to retest, **while the absolute reliability is** **the** consistency of repeated measurements for individuals across sessions.

**Hopkins WG. Measures of reliability in sports medicine and science. Sports Medicine. 2000;30:1–15.**

**We have included a brief explanation of absolute and relative reliability in methods section.**

In this part values for the tests are provided – where can the reader find the values used for the second part?

**The logarithmic transformation is a powerful technique in statistics and data analysis that helps stabilize variance and normalize distributions.**

**Again, we appreciate your comment because it is really that it is not well explained in the manuscript or can be a bit confusing. We have included the figures with the original data and we have modified the text in the manuscript explaining which is the purpose of log transformation and what is the allometry parameter (slope of the straight line of the regression with log data).**

**Now, the readers can compare the original data with the normalized data and see how the anthropometric variables influence on test performance. We have included an example in one of the plots and a practical application section to explain it and its implications for practitioners and health and sport profesionals.**

The provided data are ALL in contrast to the originally published values. This requires explanation.

**-In the section *Between-sex analysis of the isometric trunk muscle endurance test scores*, at the *Discussion* section, we showed that BST and SBT values are in line with previous originally published data except for the significant differences between males and females obtained previously in SBT. No significant differences can be related with the population characteristics, sample size, etc.**

**-Concerning the IT, which between-sex differences scores are not in line with the study of Ito et al. (25), the authors hypothesizes that they could be related to the fact that males had significantly higher mass, sitting height, biacromial breadth, and/or iliac-acromion index than females (table 2), and therefore higher gravitational extension torque during the test.**

As the Ito test originally contains a flexion and an extension part, this should be mentioned and explained. The only hint is provided in figure 1.

**- The specific Ito test used in this study has been mentioned in the manuscript:**

**“The BST (24), the IT (25) and the SBT (11) were performed to asses extensor, flexor and lateral flexor trunk endurance, respectively as described by Juan-Recio et al., (2014)”.**

**In addition, we have mentioned it in the introduction**

**“…In order to answer these questions and facilitate the use and interpretation of some of the most representative isometric trunk muscle endurance field tests [i.e., Biering-Sorensen test (BST), Abdominal Ito test (IT), Side Bridge test (SBT)]…”**

Part two (correlation calculations)
Again: what are the test data used for the calculations. Further: which anthropometric data were used? Why do the authors only provide logarithmic data? As no original data were provided, the reviewer has no chance to get an idea about the original dataset.

**The data used in the correlation analysis were the same as those used in the reliability analysis. In both analysis, data were log-transformed for both tests performance and anthropometric characteristics to ensure the homoscedasticity assumption.**

In the manuscript it is mentioned several times, that especially the upper body weight has impact on trunk muscle endurance test. Why did the authors refrain from also using this particular value? Further, the used anthropometric values are all measures in the frontal plane. But humans come as three-dimensional subjects. For me it is inconsequent to only use such parameters and not also parameters of "deepness" i.e. in the sagittal plane or, as already recommended, masses of relevant body parts.

**As you mention, an individual’s body mass and its distribution can impact performance in the isometric trunk muscle endurance test. We appreciate your suggestion and agree that it would have been better to use the actual masses of the relevant body parts. However, these measurements require an indirect and more complex calculation compared to the anthropometric measurements commonly used in this study and in previous research. (Dejanovic et al., 2012, 2014). Due to the complexity of calculating the masses of body parts, we have used anthropometric measurements related to these masses, for example: greater biacromial and biileocrestal diameters imply greater masses in the upper trunk and pelvis, respectively.**

**We have included this in the limitation section**

**Furthermore, considering the importance of body mass distribution in trunk muscle endurance performance, future studies should analyze the relationship between the masses of the relevant body parts and test scores.**

Detailed
L37-38
Not clear how many tests were performed in the mentioned sessions.

**The number of tests performed in each session have been clarify in the text:**

**“… performed the three isometric trunk holding tests twice in two testing sessions to perform the reliability analysis and later, the three tests were performed once more, but in different sessions (one for each test)…”**

L40
What do the authors mean by using the wording "relative reliability"?

**- The relative reliability represents how well the rank order of participants in one trial is replicated in the second trial and normally is expressed by ICC. We have included a brief explanation of absolute and relative reliability in *Methods* section.**

**We have included a brief explanation of absolute and relative reliability in methods section.**

L52-53
The statement about familiarization is not supported by the results

**- We have specified that some of the tests require a longer familiarization period.**

**“… but some of them require an extensive familiarization period”.**

L60
"increasing …. endurance" sounds strange as no intervention is mentioned

-We have eliminated the word “increasing” and we have rewritten the sentence introducing “…the development of trunk muscle endurance…”

L93-94
Absolute and relative reliability – not explained

**We have included a brief explanation of absolute and relative reliability in *Methods* section. In addition, the sentences include the cite of Hopkins´ study in which both are explained.**

**Hopkins WG. Measures of reliability in sports medicine and science. Sports Medicine. 2000;30:1–15.**

**We have included a brief explanation of absolute and relative reliability in methods section.**

L102
Please provide the kind of physical activity, as this might have impact on the endurance capacity

**Some examples of the kind of physical activity have been provided.**

L120-121
Eight minutes break between endurance tasks to exhaustion sound very short. How about cumulating fatigue for the three tests?

**Although rest periods of 5-10 min have been used in previous studies (Evans et al., 2007; Mcgill et al., 1999), to mitigate the effect of fatigue, the three tests were first performed in a counterbalanced way for reliability analysis, and later, the three tests were performed in separate sessions for correlation analysis.**

L124-126
No criterion mentioned to identify learning effects. Provocative question: do you think that for a simple holding test e learning effect can occur?

**- As explained in the new version of the *Discussion* section, these authors think that participants could learn small postural adjustments which could foster the alternation of activity between synergist muscles, improving the test performances. They could also improve their tolerance to the fatigue sensations that appear during these test execution. Even, the motivation for improving their test scores (common in athletes) could produce an increase in test performance.**

“… there was a significant between-session change (i.e. learning/repetition effect) in mean scores for the BST (10.56%) and the SBT (4.67%). This increase in test performance could be explained by different factors, for example: i) learning to make slight postural adjustments during the test execution that could foster the alternation of activity between synergist muscles, reducing the impact of muscle fatigue; ii) improving tolerance to the sensations of muscle fatigue (i.e. discomfort, stiffness, etc.) during test performance; and iii) the high motivation for improving test scores, common in athletes (36)”

L128
Please be consistent in citation style

**The citation style has been corrected throughout the manuscript.**

L130 – figure 1
Please provide the subfigures in the same order as described in the text. Also: just mention, that the Ito test consists of two tests: one for the abdominals and one for the back muscles. obviously here the abdominal test was used.
For the SBT slightly different times were reported per side - which side was tested here?

**The subfigures have been provided in the same order as described in the text and it has been mentioned that the IT used was for evaluating flexor endurance and that the right side of the side bridge was tested in the study.**

L143
Please define the ileospinal height (start – end)

**-The ileospinal height has been defined.**

**“…defined as the ratio between ileospinal height** (**vertical height between the anterior-superior iliac spines and the support surface**) **and body height multiplied by 100.”**

L155
Again: relative reliability… unclear.

**A brief explanation of relative reliability has been provided in the *Statistical Analysis* section	.**

L180-181
Scale of values unclear (also after reading the Hopkins paper)

**We have actualized the scale and the cite in which the scale used in this study is proposed by Hopkins.**

**Hopkins WG. Spreadsheets for analysis of validity and reliability. Sportscience. 2015;19(19):36–44.**

Results section
Please provide a clear structure, best by using subheadings – first reliability analysis and then the correlation results.

**Subheadings have been included in *Results* section.**

Table 1
Already mentioned: as the provided test times are considerably different from the original articles these data require some explanation. The term " Significant between-xxx differences" is simply wrong

**The term "Significant between-sessions differences" has been replaced by “Significant differences between sessions 1 and 2”.**

Table 2
Please provide all distance values in cm. You provide relative differences for all anthropometric variables together with typical errors and ICC values. As the reliability data are partly worse than the test reliability, this calls for an explanation.

**Authors: All distances have been provided in centimeters in table 2.**

Table 3
What is the Iliac-acromion Index? Not explained.

**The acromion-iliac index has been explained in *Methods* section.**

***“…acromion-iliac index*, defined as the ratio between the biileocrestal and biacromial breadths multiplied by 100…!**

Figures 2, 3, 4
x- slope and increments seem all to be wrong. But maybe the data are related to the "original" but not provided data.

**Authors: We have revised all x- slope and increments. These values are calculated with the log transformed data.	This procedure allows us to calculate the allometric parameter (slope of the straight line of the regression with log data. We have clarified during the manuscript (statistical analysis and results section). We appreciate your comment because it is true that it was a bit confusing. We hope it is now clearer.**

Figure 5
two linear interpolations but only one equation.... accounts for both subfigures

**Authors: After checking the multiple linear regression, we noticed that the second variable (biacromial breadth) only improved the variance explained in an 8% and in addition it was very related with the mass (collinearity). Considering this and that the normalization formula was rather more complex, we decided to remove it.**

L268-270, L293, L308-309, L347-350
Please see my comment about other promising anthropometric variables.

**We have included this in the limitation section**

**Furthermore, considering the importance of body mass distribution in trunk muscle endurance performance, future studies should analyze the relationship between the masses of the relevant body parts and test scores.**

L294-295
You state "can" - right. But be clear that a possibility is not a causality.

**Authors: We think that a larger biacromial breadth involve a larger upper-trunk mass in most cases, but not in all.	It is the reason why we write “can”.**

L298
not really, since the resulting body slope will increase together with this measure, consequently reducing the load (following the sine function). Here, body length would have been expected to be negatively correlated with holding time.

**Authors: It is true, but we think that the increase of the mass due to a greater biacromial diameter has a greater effect than the increase of the angle, approximately 0.5º considering the increase of the biacromial amplitude (2-3 cm).**

L304
What is the relationship between biileocrestal breadth and lower-trunk mass? Calls for explanation.

**We have replaced lower-trunk mass by pelvis for better clarity. As we stated in a previous sentence “…during this test execution, the pelvis is approximately in the central part of the body mass that must be held against gravity, therefore a larger biileocrestal breadth (i.e., a larger pelvis mass) also increases the gravitational torque and hinders test performance…”**

L339-340
Why do you think your results contain a learning effect? This calls for a hypothesis.

**As explained before, we think that participants could learn small postural adjustments which could foster the alternation of activity between synergist muscles, improving the test performance. They could also improve their tolerance to the fatigue sensations that appear during these test executions. Even, the motivation for improving their test scores (common in athletes) could produce an increase in test performance.**

“… there was a significant between-session change (i.e. learning/repetition effect) in mean scores for the BST (10.56%) and the SBT (4.67%). This increase in test performance could be explained by different factors, for example: i) learning to make slight postural adjustments during the test execution that could foster the alternation of activity between synergist muscles, reducing the impact of muscle fatigue; ii) improving tolerance to the sensations of muscle fatigue (i.e. discomfort, stiffness, etc.) during test performance; and iii) the high motivation for improving test scores, common in athletes (36)”

L353-358
All mentioned influencing factors were present also in the original Ito investigation. So this is no explanation.

**As we stated at the end of the paragraph…**

**“Further research is needed to explore the origin of these differences between studies, which could be related to differences in morphological characteristics, training preferences, sport practice, levels of motivation, sell-efficacy, etc. between participants”.**

L380
In all original articles data were provided separately for both sexes….

**That sentence is more aimed to health and sport professionals during their practice in sport and clinical settings than to researchers. We have specified it in the manuscript.**

**“Finally, sex-related differences in trunk endurance scores should also be considered when using these tests in sport and clinical settings, especially in cases in which there are important anthropometric differences between males and females.”**
