# Supplementary material for: The impact of anthropometric characteristics on isometric trunk muscle endurance tests: A reliability and performance analysis
Source: PLoS One. 2025 Jun 3;20(6):e0324787. doi: 10.1371/journal.pone.0324787 (PMC12132975; doi:10.1371/journal.pone.0324787)
Supplement: Attachment — Submitted filename: Plos_Rev.docx [file pone.0324787.s002.docx]

**Review of the Manuscript: Anthropometric Characteristics Impact the Participant’s Performance in Popular Isometric Trunk Muscle Endurance Tests**

**General Evaluation**

The manuscript investigates the relationship between anthropometric characteristics and performance in isometric trunk muscle endurance tests, a relevant topic in sports sciences and rehabilitation. The study is well-structured, and the methodology is clear. However, several issues require revision before publication. These include missing details on sample selection, statistical power, and methodological explanations. Furthermore, the discussion needs stronger connections to practical applications, and some statistical choices require further justification. Below is a detailed breakdown of these concerns.

**1. Title and Abstract**

**Title Revision**

- The current title is clear but could be more specific in highlighting both reliability and performance analysis. A suggestion: **"The Impact of Anthropometric Characteristics on Isometric Trunk Muscle Endurance Tests: A Reliability and Performance Analysis"**
- This version clarifies the dual focus on reliability and performance.

**Abstract (Lines 31-54)**

- The abstract effectively summarizes the study but lacks a strong emphasis on practical applications. Consider adding a concluding statement addressing how the findings can be utilized in sports science, rehabilitation, and clinical settings.
- The phrase **"some of them require an extensive familiarization period"** (Line 53) should be revised to indicate which tests specifically require longer familiarization.
- The methodological details about the **log transformation** applied to statistical data should be mentioned briefly to avoid confusion later in the text.

**2. Introduction**

**Lack of Explicit Hypotheses (Line 97)**

- While the study’s objectives are outlined, explicit hypotheses are missing. The manuscript should clearly state:
  1. **"We hypothesize that individuals with greater body mass and broader anthropometric dimensions will exhibit lower endurance test performance due to increased gravitational torque."**
  2. **"Sex-based differences will influence test performance, with females potentially showing higher endurance in certain tests due to differences in muscle fiber composition and fat distribution."**
- This addition enhances clarity and scientific rigor.

**Recent Literature Missing**

- The introduction references studies up to 2018, but more recent literature should be incorporated to support the research gap. Specifically:
  - **Recent systematic reviews (2020-present) on core endurance and its relation to anthropometric factors** should be discussed.
  - If there are any recent studies evaluating **plank or Biering-Sorensen tests in specific populations**, they should be included.

**3. Methods**

**Sample Description (Lines 100-107)**

- The manuscript states that participants were "physically active individuals," but this description is vague.
- Key missing details:
  - Were the participants **trained athletes, recreational athletes, or untrained individuals**?
  - What was the criterion for being considered "physically active"? (e.g., self-reported activity levels, validated scales like IPAQ)
  - **Was the sample size predetermined through power analysis?** (Only a post hoc analysis is provided.)
- Suggested revision:
  - **"Participants were recruited based on self-reported physical activity of at least 120–300 minutes per week of moderate exercise, but no structured core training."**

**Testing Procedures (Lines 120-121)**

- The **8-minute rest period** between endurance tasks is questionable.
  - Is this rest period sufficient to prevent residual fatigue from influencing subsequent test performance?
  - Previous studies have used 10–15 minutes of rest in similar protocols.
  - It would be beneficial to cite literature justifying the chosen rest duration.

**Reliability of Anthropometric Measurements (Line 155)**

- The manuscript does not report **inter-rater or intra-rater reliability** for anthropometric assessments.
  - Were measurements taken by the same investigator?
  - If not, was reliability assessed between raters?
  - These details are crucial to ensuring measurement consistency.

**4. Results**

**Learning Effect and Test Repetitions (Lines 339-340)**

- The study suggests a learning effect for BST and SBT. However, no **comparative analysis** is presented to confirm this.
  - A **comparison of first vs. last trial performances across all tests** should be included to validate the learning effect hypothesis.
  - Were participants given additional instructions before later trials that could have influenced their improvement?

**Statistical Power and Sample Size Justification (Lines 272-274)**

- A post hoc power analysis is provided, but no **a priori power analysis** justifying the initial sample size is presented.
  - Without an a priori power analysis, it is unclear if the sample size was adequate to detect meaningful effects.
  - The manuscript should clarify:
    - **How was the number of 45 participants determined?**
    - **Would a larger sample have produced more generalizable results?**

**Correlation Analysis Interpretation (Table 3, Lines 210-218)**

- The negative correlations between body mass and endurance scores are well discussed, but **the physiological mechanisms need further explanation**:
  - **Why does body mass negatively affect endurance?** Is it due to increased gravitational force or metabolic inefficiency?
  - **Why does the acromion-iliac index correlate with lower endurance?** What biomechanical factors contribute to this?
- The discussion should **incorporate muscle function, biomechanical load distribution, and physiological adaptations**.

**5. Discussion and Conclusion**

**Real-World Application of Findings (Lines 353-358)**

- The conclusion should provide a clearer **practical takeaway** for trainers, clinicians, and researchers.
- For example:
  - How can sports scientists apply this research in **athletic screening**?
  - How should clinicians **interpret endurance test results for rehabilitation patients**?
- Suggested addition: **"Trainers and clinicians should consider individual anthropometric differences when interpreting test results, as larger body mass and upper body breadth may artificially lower endurance scores. Adjustments to normative values may be required in applied settings."**

**Limitations and Future Directions**

- The manuscript lacks an explicit mention of key limitations:
  - **Generalizability:** Findings are limited to a specific population of young, university students.
  - **Body Composition Variables:** Fat mass and lean mass, which could influence endurance performance, were not measured.
  - **No Direct Measurement of Muscle Activation:** Surface EMG or other biomechanical analyses could enhance understanding.
- The authors should propose **future research directions**, such as:
  - Investigating how **specific training adaptations** affect endurance test results.
  - Examining endurance performance in **older adults or elite athletes**.

**6. Language and Writing**

**Grammar and Clarity**

- The manuscript is generally well-written but contains minor language errors.
- Example corrections:
  - **Line 377:** "specially" → should be "especially."
  - **Line 60:** "increasing endurance" → should be "developing endurance" (as no intervention was conducted).
  - **Line 52-53:** The claim that tests require "extensive familiarization" is **unsupported by results** and should be rewritten as:
    - **"Our results suggest that some of these tests may require longer familiarization for accurate performance assessment."**
- Though the manuscript has been proofread, a final language revision is recommended to improve clarity and precision.
