# Supplementary material for: The impact of anthropometric characteristics on isometric trunk muscle endurance tests: A reliability and performance analysis
Source: PLoS One. 2025 Jun 3;20(6):e0324787. doi: 10.1371/journal.pone.0324787 (PMC12132975; doi:10.1371/journal.pone.0324787)
Supplement: Attachment — Submitted filename: Response to Reviewers_Def.docx [file pone.0324787.s004.docx]

The authors would like to thank the Reviewers and Editors for their advice and recommendations. We believe that the manuscript is stronger as a result of their comments.

The authors’ responses to each comment are bolded, and changes in the manuscript are presented in red.

**Review of the Manuscript: Anthropometric Characteristics Impact the Participant’s Performance in Popular Isometric Trunk Muscle Endurance Tests**

**General Evaluation**

The manuscript investigates the relationship between anthropometric characteristics and performance in isometric trunk muscle endurance tests, a relevant topic in sports sciences and rehabilitation. The study is well-structured, and the methodology is clear. However, several issues require revision before publication. These include missing details on sample selection, statistical power, and methodological explanations. Furthermore, the discussion needs stronger connections to practical applications, and some statistical choices require further justification. Below is a detailed breakdown of these concerns.

**1. Title and Abstract**

**Title Revision**

- The current title is clear but could be more specific in highlighting both reliability and performance analysis. A suggestion: **"The Impact of Anthropometric Characteristics on Isometric Trunk Muscle Endurance Tests: A Reliability and Performance Analysis"**
- This version clarifies the dual focus on reliability and performance.

**Authors: We have changed the title in the manuscript**

**The Impact of Anthropometric Characteristics on Isometric Trunk Muscle Endurance Tests: A Reliability and Performance Analysis**

**Abstract (Lines 31-54)**

- The abstract effectively summarizes the study but lacks a strong emphasis on practical applications. Consider adding a concluding statement addressing how the findings can be utilized in sports science, rehabilitation, and clinical settings.
- The phrase **"some of them require an extensive familiarization period"** (Line 53) should be revised to indicate which tests specifically require longer familiarization.

**Authors: We have indicated which tests require a specifically longer familiarization period and we have included a concluding statement addressing how the findings can be utilized in sports science, rehabilitation, and clinical settings.**

**“In conclusion, trainers and clinicians should consider individual anthropometric and sex differences when interpreting test results, as a larger body mass and upper body breadth may artificially lower endurance scores. Adjustments to normative values may be required in applied settings. Moreover, based on the reliability analysis, these tests could be used to classify participants consistently,** **but the BST and the SBT require an extensive familiarization period”**

- The methodological details about the **log transformation** applied to statistical data should be mentioned briefly to avoid confusion later in the text.

**Authors: We have mentioned the methodological details about the log transformation applied to statistical data briefly.**

**To ensure the normality and homoscedasticity assumption, data (i.e., test scores and anthropometric variables) were logarithmically transformed.**

**2. Introduction**

**Lack of Explicit Hypotheses (Line 97)**

- While the study’s objectives are outlined, explicit hypotheses are missing. The manuscript should clearly state:
  1. **"We hypothesize that individuals with greater body mass and broader anthropometric dimensions will exhibit lower endurance test performance due to increased gravitational torque."**
  2. **"Sex-based differences will influence test performance, with females potentially showing higher endurance in certain tests due to differences in muscle fiber composition and fat distribution."**
- This addition enhances clarity and scientific rigor.

**Authors: We have included both hypothesis in the manuscript.**

**“Based on previous studies (12–18), we hypothesize that individuals with greater body mass and broader anthropometric dimensions will exhibit lower endurance test performance due to increased gravitational torque. In addition, sex-based differences will influence test performance, with females potentially showing higher endurance in certain tests due to differences in muscle fiber composition and fat distribution.”**

**Recent Literature Missing**

- The introduction references study up to 2018, but more recent literature should be incorporated to support the research gap. Specifically:
  - **Recent systematic reviews (2020-present) on core endurance and its relation to anthropometric factors** should be discussed.
  - If there are any recent studies evaluating **plank or Biering-Sorensen tests in specific populations**, they should be included.

**Authors: We have included two recent cites which related BMI with the Biering-Sorensen test and the 60º flexion test, but despite our best efforts we have not found any recent systematic reviews (2020-present) on core endurance and its relation to anthropometric factors in scientific literature.**

**Aydoğan K, Kostanoğlu A, Törpü GC. Effect of Body Mass Index on Balance, Trunk Muscle Endurance, Functional Mobility and, Physical Activity in College Students. Bezmialem Sci. 2024;12(4):441–9.**

**Vlažná D, Krkoška P, Kuhn M, Dosbaba F, Batalik L, Vlčková E, et al. Assessment of Lumbar Extensor Muscles in the Context of Trunk Function, a Pilot Study in Healthy Individuals. Appl Sci 2021;11(20):9518.**

**3. Methods**

**Sample Description (Lines 100-107)**

- The manuscript states that participants were "physically active individuals," but this description is vague.
- Key missing details:
  - Were the participants **trained athletes, recreational athletes, or untrained individuals**?
  - What was the criterion for being considered "physically active"? (e.g., self-reported activity levels, validated scales like IPAQ)
  - **Was the sample size predetermined through power analysis?** (Only a post hoc analysis is provided.)
- Suggested revision:
  - **"Participants were recruited based on self-reported physical activity of at least 120–300 minutes per week of moderate exercise, but no structured core training."**

**Authors: We have changed the participants’ description in the abstract.**

**“Forty-five recreational athletes (27 males and 18 females) performed the three isometric trunk holding tests”**

**In the participants section, we included that participants were recreational athletes and that they completed a questionnaire about their medical and athletic history to evaluate their health status and regular physical activity.**

**“****They were recreational athletes (i.e. practicing soccer, gymnastics, basketball, running, etc.), with a regular practice of 60-120 min of moderate to intense physical activity 2-5 times a week (for a total of 120-300 min per week, approximately). They completed a questionnaire about their medical and athletic history to evaluate their health status and regular physical activity.”**

**Testing Procedures (Lines 120-121)**

- The **8-minute rest period** between endurance tasks is questionable.
  - Is this rest period sufficient to prevent residual fatigue from influencing subsequent test performance?
  - Previous studies have used 10–15 minutes of rest in similar protocols.
  - It would be beneficial to cite literature justifying the chosen rest duration.

**Authors: We agree with the reviewer. Although rest periods of 5-10 min have been used in previous studies (Chan et al., 2005; Evans et al., 2007; McGill et al., 1999), the three tests were performed first in a counterbalanced way to mitigate the effect of fatigue in our study for reliability analysis, and later, the three tests were performed in separate sessions for correlation analysis.**

**-Chan RH. Endurance times of trunk muscles in male intercollegiate rowers in Hong Kong. Arch Phys Med Rehabil. 2005 Oct;86(10):2009-12. doi: 10.1016/j.apmr.2005.04.007. PMID: 16213246.**

**-** **Evans K, Refshauge KM, Adams R. Trunk muscle endurance tests: reliability, and gender differences in athletes. J Sci Med Sport. 2007 Dec;10(6):447-55. doi: 10.1016/j.jsams.2006.09.003. Epub 2006 Dec 1. PMID: 17141568.**

**-McGill SM, Childs A, Liebenson C. Endurance times for low back stabilization exercises: clinical targets for testing and training from a normal database. Arch Phys Med Rehabil. 1999 Aug;80(8):941-4. doi: 10.1016/s0003-9993(99)90087-4. PMID: 10453772.**

**Authors: We have included these references in the manuscript when we describe the rest period.**

**Reliability of Anthropometric Measurements (Line 155)**

- The manuscript does not report **inter-rater or intra-rater reliability** for anthropometric assessments.
  - Were measurements taken by the same investigator?
  - If not, was reliability assessed between raters?
  - These details are crucial to ensuring measurement consistency.

**Authors: The measurements were taken by the same investigator: The intra-rater relative reliability of anthropometric measurements were reported in tables 1 and 2. We have specified this in the table caption and the statistical analysis.**

**4. Results**

**Learning Effect and Test Repetitions (Lines 339-340)**

- The study suggests a learning effect for BST and SBT. However, no **comparative analysis** is presented to confirm this.
  - A **comparison of first vs. last trial performances across all tests** should be included to validate the learning effect hypothesis.
  - Were participants given additional instructions before later trials that could have influenced their improvement?

**Authors: This paragraph has been included in statistical analysis section.**

**“A two-way ANOVA was conducted to determine the differences between the two first testing sessions (learning effect) and sex: 3 (IT, BST, SBT) x 2 (session 1, session 2) x 2 (males, females). If significant differences were identified, the Bonferroni post hoc analysis was applied for pairwise comparisons.”**

**We analyzed the reliability in the first two sessions in which all the tests were performed together. The examiner gave the same instructions during both sessions and the participants were encouraged to maintain the postures for as long as possible when they started to show signs of fatigue.**

**Statistical Power and Sample Size Justification (Lines 272-274)**

- A post hoc power analysis is provided, but no **a priori power analysis** justifying the initial sample size is presented.
  - Without an a priori power analysis, it is unclear if the sample size was adequate to detect meaningful effects.
  - The manuscript should clarify:
    - **How was the number of 45 participants determined?**
    - **Would** **a larger sample have produced more generalizable results?**

**Authors: We carried out a priori power analysis considering a *r*= -0.55 (Nuzzo and Mayer, 2013) and an α= 0.05; 1–β= 0.80. The total sample size calculated was 16 participants (32 participants considering males and females).**

**Because a few correlations obtained in this study were under 0.55 we considered more appropriate that including a post-hoc power analysis in order to show the real statistical power of this study was more appropriate. In the limitation section, we suggested that future studies should perform correlation analysis with larger samples in different populations:**

**Study limitations**

These findings are limited to a specific population of young university students. Moreover, since a larger sample would have allowed more generalizable results, **future studies should perform correlational analysis with larger samples in different populations (e.g., sedentary people, high performance athletes, patients, older adults, etc.), which would allow: i)** **to understand which anthropometric variables have a greater influence on the test scores better; ii) to develop much more accurate predictive models and; and iii) to elaborate** **normalized normative data that would allow to categorize the physical condition of the participants in a more realistic and adjusted way.**

**Correlation Analysis Interpretation (Table 3, Lines 210-218)**

- The negative correlations between body mass and endurance scores are well discussed, but **the physiological mechanisms need further explanation**:
  - **Why does body mass negatively affect endurance?** Is it due to increased gravitational force or metabolic inefficiency?
  - **Why does the acromion-iliac index correlate with lower endurance?** What biomechanical factors contribute to this?
- The discussion should **incorporate muscle function, biomechanical load distribution, and physiological adaptations**.

**Authors: We have included the explanation why body mass negatively affect endurance**

“A greater body mass implies an increased gravitational force which negatively affects trunk endurance”

**In the discussion section we discussed why the acromion-iliac index correlates with lower endurance**

**“In this sense, a trapezoidal trunk (i.e. an acromion-iliac index below 69.9) or short limbs (i.e. a relative length of the lower limbs below 54.9) (25), as is the case in our study (acromion-iliac index in males: 68.13±4.78; relative length of the lower limbs in females: 54.77±1.64), also represent a larger upper-trunk mass and a possible disadvantage for these holding test execution. In the SBT the upper-trunk mass has a single-upper limb support, so a larger biacromial breadth implies greater forces on the shoulder, elbow and forearm of the lowermost side and higher difficulty in maintaining the posture. In this sense, Juan-Recio et al., (17) showed that the shoulder muscle activation and the individuals’ anthropometric characteristics played an important role in the SBT performance”.**

**5. Discussion and Conclusion**

**Real-World Application of Findings (Lines 353-358)**

- The conclusion should provide a clearer **practical takeaway** for trainers, clinicians, and researchers.
- For example:
  - How can sports scientists apply this research in **athletic screening**?
  - How should clinicians **interpret endurance test results for rehabilitation patients**?
- Suggested addition: **"****Trainers and clinicians should consider individual anthropometric differences when interpreting test results, as larger body mass and upper body breadth may artificially lower endurance scores. Adjustments to normative values may be required in applied settings."**

**Limitations and Future Directions**

- The manuscript lacks an explicit mention of key limitations:
  - **Generalizability:** Findings are limited to a specific population of young, university students.
  - **Body Composition Variables:** Fat mass and lean mass, which could influence endurance performance, were not measured.
  - **No Direct Measurement of Muscle Activation:** Surface EMG or other biomechanical analyses could enhance understanding.
- The authors should propose **future research directions**, such as:
  - Investigating how **specific training adaptations** affect endurance test results.
  - Examining endurance performance in **older adults or elite athletes**.

**Authors: We have included the suggestions of the reviewer in the study limitations section.**

**6. Language and Writing**

**Grammar and Clarity**

- The manuscript is generally well-written but contains minor language errors.
- Example corrections:
  - **Line 377:** "specially" → should be "especially."
  - **Line 60:** "increasing endurance" → should be "developing endurance" (as no intervention was conducted).
  - **Line 52-53:** The claim that tests require "extensive familiarization" is **unsupported by results** and should be rewritten as:
    - **"Our results suggest that some of these tests may require longer familiarization for accurate performance assessment."**
- Though the manuscript has been proofread, a final language revision is recommended to improve clarity and precision.

**Authors: We have revised all language errors and we have changed “increasing” by “development”.**

**Considering the fact that trunk muscle endurance deficits and imbalances have been related with low back pain (1–3) and that trunk muscle fatigue has a harmful effect in muscular coordination, postural control and spine stability (4,5), the development of trunk muscle endurance is a common training program goal for injury treatment and prevention, functional capacity improvement in daily tasks and sport performance (6–8).**

**As we stated in the statistical analysis an ANOVA was conducted to determine the differences between the two first testing sessions. In addition, in the results section we indicate that “The ANOVA showed a significant effect in the within-subject factor “session” for the BST (*p*≤ .001; F= 15.743) and the SBT (*p*= .038; F= 4.58), but not in the session*sex interaction in any of the tests (*p*> .050).”**
